# svZeroDSolver: A modular package for lumped-parameter cardiovascular simulations

**DOI:** 10.21105/joss.07595

**Published:** 2025-05-23

**Authors:** Karthik Menon, Jakob Richter, Martin R. Pfaller, Jonathan Pham, Emilin M. Mathew, Kaitlin E. Harold, Nicholas C. Dorn, Aekaansh Verma, Alison L. Marsden

**Affiliations:** 1 Georgia Institute of Technology, Atlanta, GA, United States of America; 2 Stanford University, Stanford, CA, United States of America; 3 Yale University, New Haven, CT, United States of America

## Abstract

Computational modeling of cardiovascular blood flow has emerged as a valuable tool in the diagnosis and treatment of cardiovascular disease (Menon, Hu, et al., 2024). While simulations of blood flow can be performed using high and low-fidelity techniques, lumped-parameter or zero-dimensional modeling is a widely used low-order technique in applications which require quick estimation of bulk flow quantities, such as flow and pressure at specific anatomical locations (Pfaller et al., 2024).

We introduce svZeroDSolver, an efficient and modular package for performing lumped-parameter (zero-dimensional) simulations of cardiovascular blood flow. As part of the SimVascular open-source project, svZeroDSolver and SimVascular together allow users to go from medical imaging to fast zero-dimensional evaluations of patient-specific hemodynamics. svZeroDSolver is written in C++ using a modular object-oriented framework. Simply specifying a .json dictionary of lumped-parameter “blocks” – such as blood vessels, junctions between blood vessels, and boundary conditions (along with their associated parameters) – allows the code to automatically assemble and solve the governing equations corresponding to the user-specified vascular model. In addition, the package includes Python and C++ interfaces to facilitate its use with other software packages. For example, it can be integrated into Python-based optimization and uncertainty quantification applications (Lee et al., 2024; Menon, Zanoni, et al., 2024; Richter et al., 2025; Zanoni et al., 2024). It can also be interfaced with C++/Fortran software for high-fidelity cardiovascular flow simulations, where svZeroDSolver can conveniently provide physiological lumped-parameter boundary conditions (Menon et al., 2023; Menon, Khan, et al., 2024). svZeroDSolver includes an application called svZeroDCalibrator to automatically calibrate parameters of a given zero-dimensional model based on independent hemodynamic measurements or high-fidelity simulations – thus improving the accuracy of zero-dimensional models (Richter et al., 2025). It also includes graphical interfaces to interactively create lumped-parameter models for simulations, as well as to visualize the simulated anatomy and hemodynamics.

## Statement of need

Non-invasive quantification of patient-specific hemodynamics via computational simulations has improved patient outcomes and reduced invasive clinical procedures in large randomized clinical trials (Taylor et al., 2023). Computational modeling is also a promising tool for non-invasive and personalized optimization of clinical treatments and surgery (Marsden, 2014).

Previous work has used several techniques to model cardiovascular blood flow, all of which can be broadly categorized based on their level of fidelity. High-fidelity models generally involve simulations of the full three-dimensional flow field within anatomical regions of interest (Menon, Hu, et al., 2024; Updegrove et al., 2017). While these are the most accurate and informative, they are computationally expensive (each simulation can take several hours or days on hundreds of CPU cores) and, therefore, not practical in typical clinical settings or for applications such as optimization and uncertainty quantification, which often require thousands of model evaluations. On the other end of the spectrum, lumped-parameter or zero-dimensional models provide information about bulk hemodynamics, such as flow rate and pressure, at specific anatomical regions of interest. While these models are not spatially resolved, they are valuable in applications which require near real-time quantification of bulk hemodynamics, as well as those that rely on thousands of repeated model evaluations (Lee et al., 2024; Menon, Zanoni, et al., 2024; Richter et al., 2025; Zanoni et al., 2024). They are also commonly used in conjunction with high-fidelity simulations where lumped-parameter models are used as physiological boundary conditions (Menon et al., 2023; Menon, Khan, et al., 2024).

svZeroDSolver, which is a part of the SimVascular open-source project, is a new open-source software package that enables fast evaluation of zero-dimensional hemodynamics. One major challenge in zero-dimensional modeling that svZeroDSolver addresses is that different clinical applications (and individual clinical cases within the same application) often require unique anatomical arrangements of blood vessels, heart valves, etc. Moreover, distinct anatomical configurations are governed by a distinct set of governing equations. Therefore, it is common for users to implement application-specific solvers which simulate the equations governing a specific application or anatomical configuration. In contrast, the modularity of svZeroDSolver allows users to easily create arbitrary anatomical configurations by arranging a library of available “blocks”, following which the software automatically assembles the equations governing the user-specified configuration.

Another unique feature of svZeroDSolver is its ability to easily interface with other C++ and Python packages. This has been used in previous work on uncertainty quantification (Lee et al., 2024; Menon, Zanoni, et al., 2024; Richter et al., 2025; Zanoni et al., 2024) as well as in multi-scale simulations coupling three-dimensional hemodynamics with zero-dimensional representations of downstream circulation (Menon et al., 2023; Menon, Khan, et al., 2024). The C++ interface has been coupled with the high-fidelity multi-physics solver svFSIplus, which is part of the widely used SimVascular open-source software project for cardiovascular biomechanics simulations (Updegrove et al., 2017; Zhu et al., 2022). svZeroDSolver has also been integrated into the graphical user interface of the SimVascular project. This allows users to leverage the functionality in SimVascular to generate three-dimensional patient-specific anatomical models from medical images and subsequently perform patient-specific zero-dimensional simulations of blood flow by automatically converting the three-dimensional anatomy into a zero-dimensional model (Pfaller et al., 2022). The automatic conversion of arbitrary patient-specific anatomies to zero-dimensional simulations is possible due to the modular nature of svZeroDSolver. Using this pipeline, previous work has demonstrated accelerated convergence of three-dimensional simulations when using corresponding zero-dimensional simulation results as initial conditions (Pfaller et al., 2021).

In addition, svZeroDSolver includes several applications to augment its functionality. The svZeroDCalibrator application improves the accuracy of zero-dimensional models by optimizing the parameters of blood vessels to recapitulate observed hemodynamics from measurements or high-fidelity simulations. This allows users to build more accurate zero-dimensional models than those typically based purely on the anatomy of the vascular region of interest (Richter et al., 2025). The svZeroDGUI application is a web-based graphical interface that allows users to create zero-dimensional simulations by interactively dragging and dropping individual blood vessels, heart chambers, boundary conditions, connections between these blocks, etc. Another graphical application, svZeroDVisualization, is an interface to visualize the lumped-parameter structure of given anatomical models as well as the simulated hemodynamics within each block. Together, these graphical interfaces make svZeroDSolver intuitive for a wide range of users, potentially expanding its use from research to instructional and clinical contexts. The functionality and accuracy of svZeroDSolver is assessed using continuous integration tests on GitHub, and has also been verified by comparing with high-fidelity three dimensional simulations (Pfaller et al., 2022). This combination of features makes svZeroDSolver uniquely applicable to a wide range of applications in cardiovascular biomechanics.

## State of the field

While there are other open-source projects that provide the functionality for cardiovascular flow modeling, and specifically zero-dimensional flow modeling, svZeroDSolver has several features that distinguish it from previous work. In particular, prior packages have primarily focused on multi-physics finite element modeling for cardiovascular biomechanics (Africa et al., 2024; Arthurs, 2021; Hirschvogel, 2024; Zhu et al., 2022). Although these projects allow the implementation of simple zero-dimensional models, either as boundary conditions to three-dimensional models or as simple stand-alone zero-dimensional models, the primary focus is on the modeling of full three-dimensional fluid and tissue mechanics. Due to this, they generally lack the variety and/or modular functionality to create a broad range of user-specified zero-dimensional flow models. There are, however, packages aimed specifically at reduced-order modeling for cardiovascular flows. For example, the SimVascular project includes svOneDSolver for the purpose of one-dimensional blood flow modeling. Another popular package for one-dimensional blood flow simulations is Nektar1D (Alastruey et al., 2012). Similarly, Artery.FE implements one-dimensional blood flow modeling using the FEniCS finite element framework (Agdestein et al., 2018), the VaMpy toolkit includes a package for modeling one-dimensional blood flow using the Lax-Wendroff finite difference method (Diem & Bressloff, 2017), and openBF is a finite volume implementation of one-dimensional blood flow (Benemerito et al., 2024).

In the zero-dimensional modeling context, CRIMSON (Arthurs, 2021), lifex-cfd (Africa et al., 2024), and Ambit (Hirschvogel, 2024) include the ability to simulate simple zero-dimensional blood flow models. However, as mentioned above, their focus is on multi-physics simulations of cardiovascular biomechanics. Therefore, they support a limited set of stand-alone zero-dimensional models and do not feature the modularity that enables the creation of a large variety of zero-dimensional models as in svZeroDSolver. The CellML and CVSim packages include a limited set of stand-alone zero-dimensional flow models for specific anatomies/applications (Clerx et al., 2020; Heldt et al., 2010), but they do not provide the modular functionality to specify unique anatomical models. In addition, there have been other packages that use zero-dimensional modeling techniques with a focus on statistical analysis, cardiac electromechanics, or specific anatomical models (Huttary et al., 2017; Regazzoni & Quarteroni, 2021; Rosalia et al., 2021). However, these packages are either not focused on zero-dimensional modeling or use MATLAB implementations, which require software licenses and are not free to use.

In contrast to these existing packages, the purpose of svZeroDSolver is to provide an open-source framework specifically for simulating zero-dimensional flows in a variety of simple and complex anatomies that can be designed in a user-specific and application-specific manner – by leveraging the modular nature of the code. The unique features listed above allow the use of svZeroDSolver both as a stand-alone zero-dimensional flow solver for unique and patient-specific anatomies, as well as in conjunction with the aforementioned multi-physics solvers as boundary conditions, for parameters estimation and uncertainty quantification, or even as an instructional tool using its graphical interfaces.

## Software details

**Figure 1:**
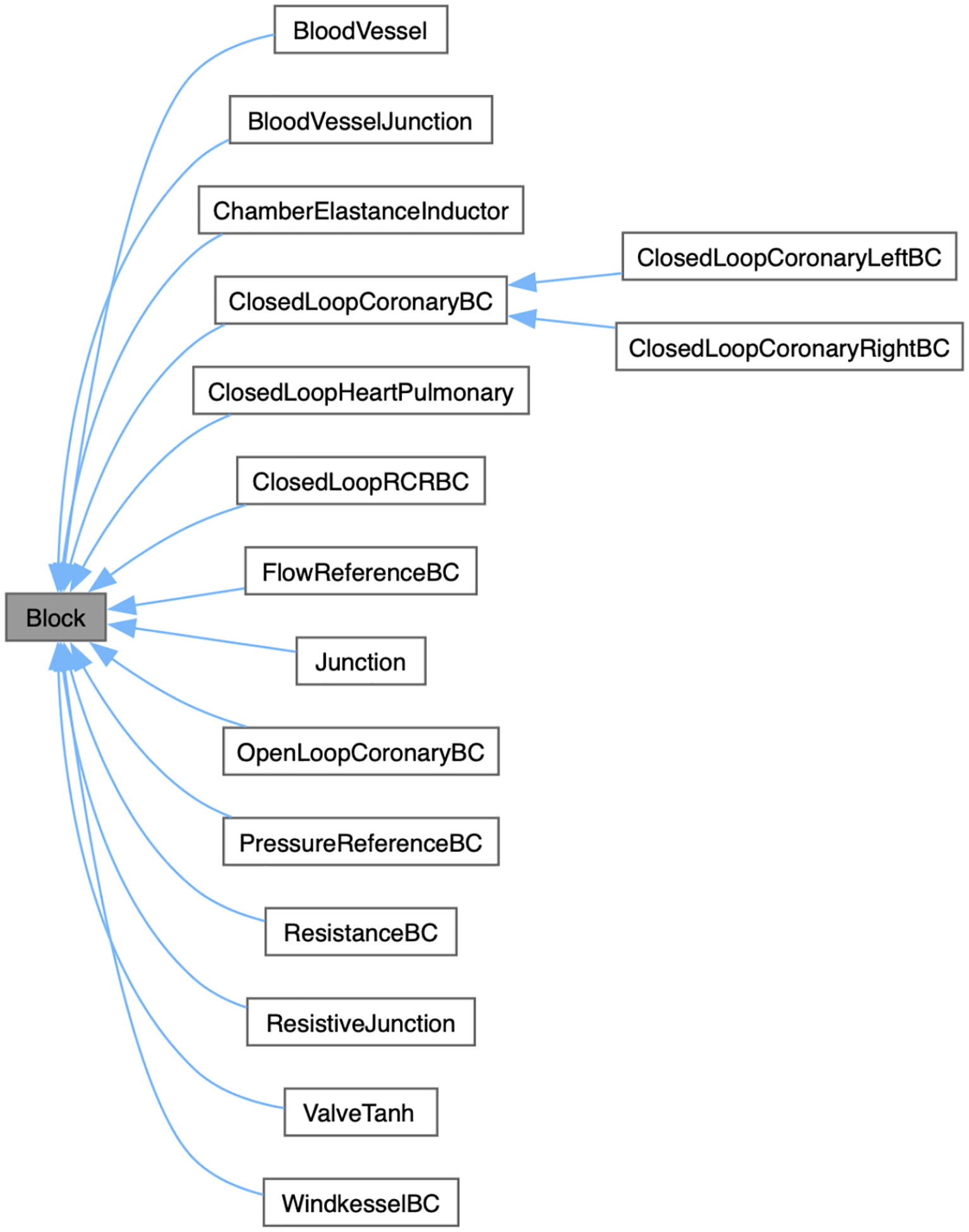
Various zero-dimensional “blocks” included in svZeroDSolver at the time of writing.

svZeroDSolver relies on a collection of “blocks” to set up the governing equations for a given anatomical configuration. Each block is inherited from a block class, as illustrated in Figure 1, and is governed by a “local” set of equations with associated degrees of freedom. The solver parses through an input configuration .json file, which lists the blocks, their parameters, and the blocks’ connectivity, and then automatically assembles the local equations and degrees of freedom for each block into a global system of equations. The governing equations and circuit representation for each block are available in the documentation. For example, see the documentation for a blood vessel block.

The zero-dimensional simulations performed by svZeroDSolver are governed by non-linear differential algebraic equations. We integrate these equations in time using the implicit generalized-alpha scheme (Jansen et al., 2000) with Newton-Raphson iterations to solve the linearized system. Under the hood, these linearized governing equations for each block are implemented as local contributions to a system of linear (matrix) equations, which are then assembled into a global linear system based on the user-specified configuration. Details on the modular implementation of the blocks, along with their governing equations, are provided in the documentation’s Developer Guide. We use the Eigen package to represent and solve these sparse linear systems. Mathematical details on this implementation are provided in the SparseSystem and Integrator classes in the documentation.

svZeroDSolver currently has implementations of different types of blood vessel blocks with non-linear resistors to model vascular stenoses, junctions between blood vessels, a heart valve block modeled using a hyperbolic tangent function, a cardiac chamber block modeled as a time-varying capacitor and inductor, and several boundary condition blocks including simple flow, pressure and resistors blocks, windkessel boundary conditions, coronary boundary conditions that include the intramyocardial pressure experienced by coronary arteries, as well as two-sided versions of windkessel and coronary boundary conditions that allow a user to build closed-loop circulation models (H. Kim et al., 2010; H. J. Kim et al., 2009; Menon et al., 2023; Menon, Khan, et al., 2024; Mirramezani et al., 2019; Vignon-Clementel et al., 2006). The input to svZeroDSolver is a .json file which specifies the simulation parameters (number of time steps, cardiac cycles, etc.), the types of blocks to be included in the specific model, the boundary conditions, and how the blocks are connected (typically using junction blocks). Each of these blocks generally requires several parameters, which can be specified using a steady value or a list of time-varying values. The solver can either run simulations for a specified number of time steps and cardiac cycles or until the difference in mean quantities between consecutive cardiac cycles is below a given threshold.

The documentation for svZeroDSolver is automatically built on GitHub using Doxygen. It includes instructions for installation, user guides for svZeroDSolver and its various applications, as well as mathematical and graphical descriptions of each zero-dimensional block that is implemented in the solver. Examples of configuration files to run svZeroDSolver simulations using the various available blocks are in svZeroDSolver/tests/cases. The repository also includes examples demonstrating the simple API for interfacing between svZeroDSolver and external C++ software packages in svZeroDSolver/tests/test_interface. Details on creating zero-dimensional simulations from three-dimensional models using the SimVascular graphical interface are available in the SimVascular documentation.

Future development plans include functionality to specify time-varying block parameters as mathematical expressions using the exprtk package. We are also expanding the available blocks to more accurately model hemodynamics, such as by using data-driven models for pressure losses at arbitrarily shaped vascular junctions (Rubio et al., 2025). In addition, we plan to extend the svZeroDGUI application to interactively create custom zero-dimensional boundary conditions for three-dimensional simulations. The development team actively implements new features, blocks and test cases to build on the capabilities of svZeroDSolver and ensure its accuracy and speed.
